# Crystal Structure of *Streptococcus pyogenes* Csn2 Reveals Calcium-Dependent Conformational Changes in Its Tertiary and Quaternary Structure

**DOI:** 10.1371/journal.pone.0033401

**Published:** 2012-03-30

**Authors:** Yoon Koo, Du-kyo Jung, Euiyoung Bae

**Affiliations:** 1 Department of Agricultural Biotechnology, Seoul National University, Seoul, South Korea; 2 Center for Agricultural Biomaterials, Seoul National University, Seoul, South Korea; Helmholtz Centre for Infection Research, Germany

## Abstract

Clustered regularly interspaced short palindromic repeats (CRISPR) and CRISPR-associated (Cas) proteins constitute a microbial immune system against invading genetic elements, such as plasmids and phages. Csn2 is an Nmeni subtype-specific Cas protein, and was suggested to function in the adaptation process, during which parts of foreign nucleic acids are integrated into the host microbial genome to enable immunity against future invasion. Here, we report a 2.2 Å crystal structure of *Streptococcus pyogenes* Csn2. The structure revealed previously unseen calcium-dependent conformational changes in its tertiary and quaternary structure. This supports the proposed double-stranded DNA-binding function of *S. pyogenes* Csn2.

## Introduction

Clustered regularly interspaced short palindromic repeats (CRISPR) are a class of repetitive genetic elements found within many bacterial and archaeal genomes [1]. These elements consist of a few to hundreds of repeated DNA sequences, typically 20 to 50 base pairs long, interspersed with variable spacer sequences, some of which are identical to those of known phages and plasmids. CRISPR-associated (*cas*) genes are located adjacent to a CRISPR locus, and many Cas proteins possess motifs and/or domains related to nucleic acid binding and processing [2].

Mounting evidence indicates that CRISPR and Cas proteins represent a microbial immune system that protects against invading foreign genetic elements, such as plasmids and phages [1]. Although the detailed molecular mechanisms are not yet fully known, three distinct stages – adaptation, expression and interference – have been recognized for the immune response mediated by the CRISPR/Cas system [1], [3], [4]. In the adaptation stage, fragments of foreign nucleic acids are integrated into the host microbial genome as variable spacers. During the expression and interference processes, these spacers are transcribed and used to recognize re-invading foreign nucleic acids, leading to their degradation. The Cas proteins are involved in these three processes.

Csn2 is one of four Cas proteins that comprise the Nmeni subtype of the CRISPR/Cas system [4]. These four proteins include two universal Cas proteins (Cas1 and Cas2), and two subtype-specific Cas proteins (Csn1 and Csn2). Previous studies on the Nmeni subtype CRISPR/Cas systems in *Streptococcus pyogenes* and *Streptococcus thermophilus* indicated that Csn1, also known as Cas9 [4], is the only Cas protein in the system required for the expression and interference processes, suggesting that Csn2 participates in the adaptation stage [5], [6]. More recently, the Nmeni subtype of the CRISPR/Cas system was newly classified as a type II CRISPR/Cas system, which can be further divided into two subtypes, II-A and II-B [4]. In subtype II-B, Csn2 is replaced with Cas4, which is sometimes fused to Cas1, and Cas4 is proposed to be involved in CRISPR adaptation together with Cas1 [4], [7]. This also suggests a role for Csn2 in the adaptation stage. In a recent study, the crystal structure of *Enterococcus faecalis* Csn2 was determined to a resolution of 2.7 Å [8]. Based on the structural analysis and other biochemical experiments, the authors proposed that Csn2 binds to double-stranded DNA (dsDNA) via calcium-dependent tetramerization [8].

Here, we report the crystal structure of *S. pyogenes* Csn2 solved to a resolution of 2.2 Å. Our structure allows for a more detailed structural analysis of the Csn2 protein, and reveals a previously unseen conformational state. We found that subunits of the tetrameric arrangement display heterogeneity in calcium binding, which results in considerable conformational changes in both the tertiary and quaternary structures. Further analysis of this conformational switching suggested a role for calcium binding beyond regulating oligomerization and supported the DNA-binding function of *S. pyogenes* Csn2.

## Results and Discussion

### Double-stranded DNA binding of *S. pyogenes* Csn2

**Figure 1:**
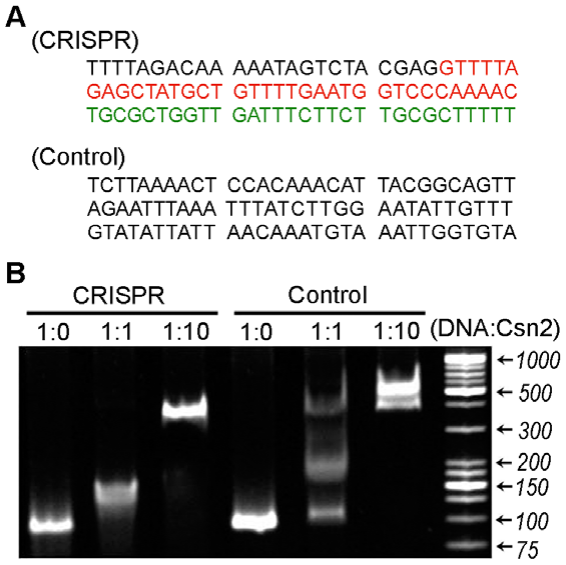
Double-stranded DNA binding of *S. pyogenes* Csn2. **A**: Sequences of *S. pyogenes* CRISPR and control DNA fragments used for an electrophoretic mobility shift assay. The repeat and the first spacer of *S. pyogenes* CRISPR are shown in red and green, respectively. The control DNA fragment contains the promoter site of the *Early Responsive to Dehydration Stress 1* gene from *A. thaliana*. **B**: An electrophoretic mobility shift assay was performed with 150 ng of dsDNA (90 bp) and increasing concentrations of *S. pyogenes* Csn2. The molar ratio of DNA to *S. pyogenes* Csn2 tetramer is indicated for each lane.

To analyze the dsDNA-binding activity of *S. pyogenes* Csn2, an electrophoretic mobility shift assay was performed using two 90-bp dsDNAs (Figure 1A). One was a section of *S. pyogenes* CRISPR DNA that included the first repeat and spacer sequences, and the other was a control DNA fragment containing the promoter site of the *Early Responsive to Dehydration Stress 1* gene from *Arabidopsis thaliana*.

The results of the mobility shift assay indicate that *S. pyogenes* Csn2 has a non-specific dsDNA-binding function (Figure 1B). The migration of the dsDNA in the gel was slower in the presence of *S. pyogenes* Csn2, and the shift was greater with the addition of more *S. pyogenes* Csn2 protein. The binding appeared to be non-specific as the shift was also observed for the control DNA, which has a completely different sequence and no relationship to the CRISPR/Cas system.

In a recent study of *E. faecalis* Csn2, it was also proposed that Csn2 protein binds to dsDNA in a non-specific fashion [8]. Considering the results from these two homologous proteins, it is likely that the physiological function of Csn2 involves the binding of dsDNA.

### Structure of *S. pyogenes* Csn2

The crystal structure of *S. pyogenes* Csn2 was determined to a resolution of 2.2 Å using multiwavelength anomalous diffraction. Data collection and refinement statistics are summarized in Table 1. The asymmetric unit of the structure contains two *S. pyogenes* Csn2 monomers, three calcium ions, 204 water molecules and two ethylene glycol molecules. Several residues (residues 40–41, 48–50, 210–213 and 220 in monomer A, and residues 40–41, 48–50, 140 and 220 in monomer B) were not included in the final model due to insufficient electron density.

**Table 1:** Data collection and refinement statistics.

	SeMet Csn2			Native Csn2
Space group	C222_1_			C222_1_
Unit cell parameters (Å)	a = 59.5, b = 163.4, c = 150.0			a = 59.1, b = 162.9, c = 149.0
	Se-peak	Se-edge	Se-remote	
Wavelength (Å)	0.97910	0.97927	0.96404	1.00000
Data collection statistics				
Resolution range (Å)	50.00-2.20 (2.28-2.20)	50.00-2.20 (2.28-2.20)	50.00-2.20 (2.28-2.20)	49.68–2.90 (3.06–2.90)
Number of reflections	36996 (3608)	37023 (3608)	37021 (3609)	16061 (2286)
Completeness (%)	98.7 (97.6)	98.7 (97.6)	98.7 (97.6)	98.4 (97.8)
R_merge_ a (%)	6.8 (65.4)	6.5 (66.6)	6.0 (68.4)	10.9 (53.5)
Redundancy	13.5 (13.2)	13.5 (13.1)	13.5 (13.2)	6.6 (6.1)
Mean I/σ	31.1 (5.1)	32.8 (4.9)	36.9 (4.8)	11.7 (2.8)
Refinement statistics				
Resolution range (Å)	29.17–2.20			49.68–2.90
Number of reflections, total/test	35414/1777			15232/813
R_cryst_ b/R_free_ c (%)	21.5/25.8			21.5/26.7
RMSD bonds (Å)	0.008			0.017
RMSD angles (deg)	1.087			1.714
Average B factor (Å^2^)	50.37			65.14
Number of water molecules	204			28
Ramachandran favored (%)	97.8			92.3
Ramachandran allowed (%)	2.2			7.7

Values in parentheses are for the highest-resolution shell.

a R_merge_ = Σ_h_Σ|I_i_(h)−<I(h)>|/Σ_h_Σ_i_I_i_(h), where I_i_(h) is the intensity of an individual measurement of the reflection and <I(h)> is the mean intensity of the reflection.

b R_cryst_ = Σ_h_||F_obs_|−|F_calc_||/Σ_h_|F_obs_|, where F_obs_ and F_calc_ are the observed and calculated structure factor amplitudes, respectively.

c R_free_ was calculated as R_cryst_ using 5.0% of the randomly selected unique reflections that were omitted from structure refinement.

**Figure 2:**
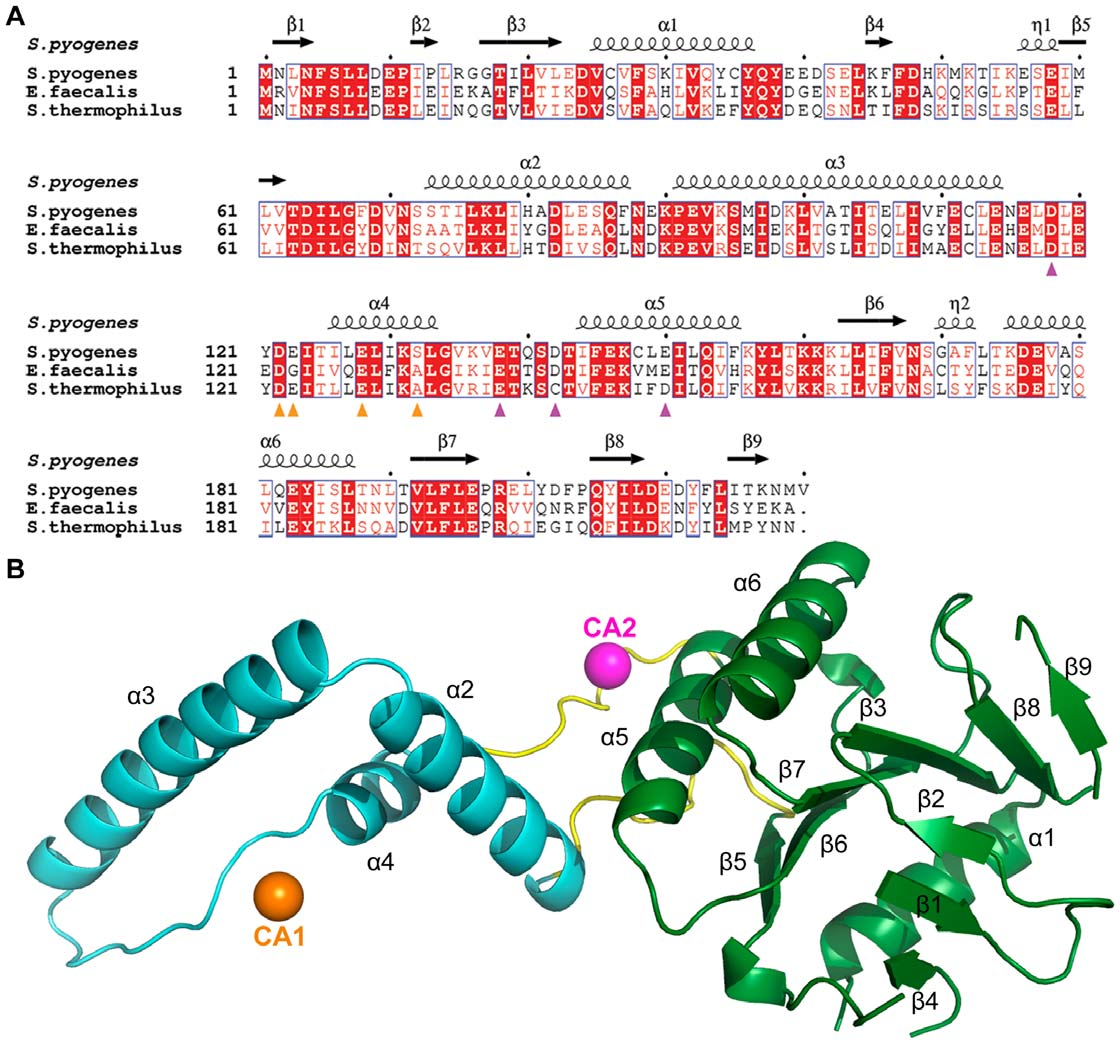
Sequence alignment and monomeric structure of *S. pyogenes* Csn2. **A**: Sequence alignment of Csn2 homologues from *S. pyogenes*, *E. faecalis* and *S. thermophilus*. Secondary structure elements are indicated based on the *S. pyogenes* Csn2 structure. The calcium coordinating residues within the CA1 and CA2 sites are marked with orange and purple triangles, respectively. **B**: Structure of *S. pyogenes* Csn2 monomer A. The globular α/β domain, the extended α-helical domain and the hinge regions are shown in green, cyan and yellow, respectively. Secondary structure elements are also indicated. Bound calcium ions in the CA1 and CA2 sites are represented as orange and purple spheres, respectively.

The monomeric structure of *S. pyogenes* Csn2 contains six α-helices (α1–α6) and nine β-strands (β1–β9), and is organized into two domains (Figure 2). The globular α/β domain (residues 1–62 and 144–219) comprises a six-stranded mixed β-sheet (β3, β5–β9), a three-stranded anti-parallel β-sheet (β1, β2, β4), and three flanking α-helices (α1, α5, α6). The mixed β-sheet is located at the center of the domain, and formed by a five-stranded parallel β-sheet (β3, β5–β8) and an additional β-strand (β9) that is aligned in the opposite direction. The convex side of the central β-sheet is flanked by two of the three α-helices (α5, α6). The α1 helix and the small three-stranded β-sheet are located on the concave side of the central β-sheet. The remaining part of the Csn2 monomer is the extended α-helical domain (residues 73–133) that protrudes from the α/β domain, and consists of three α-helices (α2–α4). The two domains are connected by two flexible hinge regions (residues 63–72 and 134–143). The α-helical domain and the hinge regions are stabilized by calcium ion binding and interaction with a symmetry-related molecule. This symmetry-related molecule is a subunit of the physiologically relevant *S. pyogenes* Csn2 tetramer (see below).

**Figure 3:**
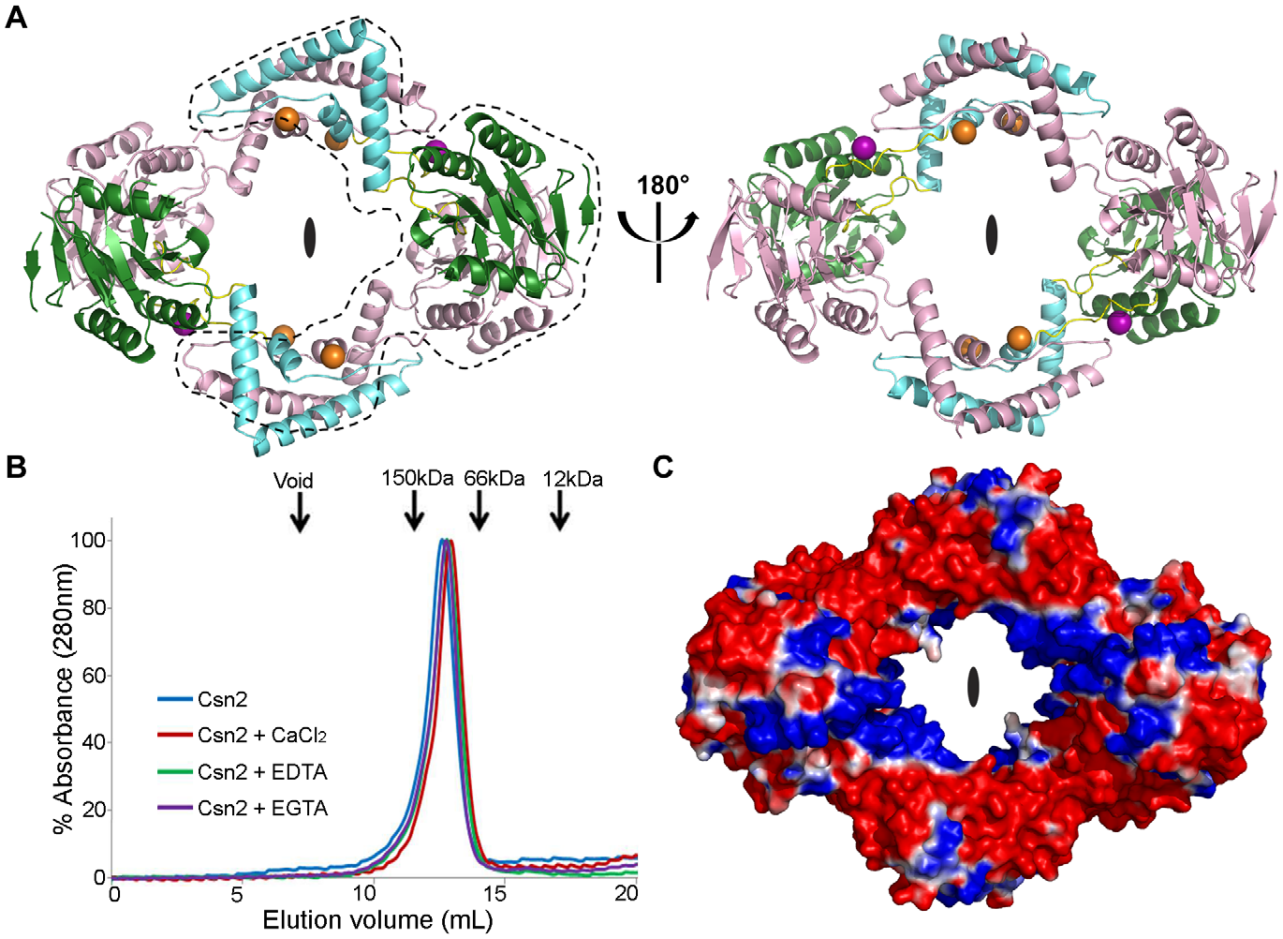
Tetrameric structure of *S. pyogenes* Csn2. **A**: Tetrameric arrangement of *S. pyogenes* Csn2. Monomer A is colored as in Figure 2B, and monomer B is shown in pink. The tetramer is viewed along the two-fold symmetry axis. The two *S. pyogenes* Csn2 monomers found in the asymmetric unit are enclosed by the black dashed line. **B**: Analytical size-exclusion chromatography of *S. pyogenes* Csn2. Elution profiles with different buffer conditions are represented by different colors. Elution volumes for molecular weight standards are also indicated. **C**: Electrostatic potential surface (red = −25 kT, blue = +25 kT) of the *S. pyogenes* Csn2 tetramer. Pymol (www.pymol.org) was used to calculate APBS electrostatics including the bound calcium ions [27].

In the asymmetric unit, the two *S. pyogenes* Csn2 monomers form a dimer with approximate two-fold non-crystallographic symmetry (Figure 3A). Only the globular α/β domain of each monomer participates in the dimerization, burying 2116 Å^2^ of total solvent accessible surface area between the two monomers and forming 9 hydrogen bonds. The dimer interface is stabilized by contacts between residues in or adjacent to α1 and the loop regions on the α5/α6 side of the central β-sheet. The extended α-helical domains of the two Csn2 monomers are not involved in internal contacts within the asymmetric unit, but participate in inter-subunit contacts with the α-helical domains of the symmetry-related dimer within the crystal lattice (Figure 3A). Monomer A in the asymmetric unit interacts with monomer B of the symmetry-related dimer, while monomer B in the asymmetric unit makes contacts with monomer A of the symmetry-related molecule. The interaction of the two α-helical domains buries 4317 Å^2^ of total solvent accessible surface area, forming 23 hydrogen bonds.

The close proximity of the α-helical domains of the two symmetry-related dimers indicates dimerization of the dimers, resulting in a *S. pyogenes* Csn2 tetramer. Analytical size-exclusion chromatography supported the tetrameric structure of the *S. pyogenes* Csn2 whose monomeric molecular weight is 26.0 kDa (Figure 3B). In the *E. faecalis* Csn2 crystal structure, the asymmetric unit contains two tetramers [8]. The tetrameric nature of *S. pyogenes* Csn2 results in a diamond-shaped ring structure with a positively charged inner surface as seen in *E. faecalis* Csn2 (Figure 3C) [8]. Based on this structural feature and other biochemical data, it was previously proposed that Csn2 may function as a dsDNA-binding protein [8].

### Calcium binding in *S. pyogenes* Csn2

We found three potential metal binding sites within the asymmetric unit of the *S. pyogenes* Csn2 structure. To reveal the identity of the bound metals, the concentrations of five metals (Ca, Mg, Mn, Co, and Ni) in *S. pyogenes* Csn2 samples were determined using inductively coupled plasma mass spectrometry (ICP-MS) and inductively coupled plasma atomic emission spectroscopy (ICP-AES). Only calcium was detected in significant amounts in the samples, and its molar quantity was comparable to that of Csn2 (Table 2). The *E. faecalis* Csn2 structure revealed calcium ions at the equivalent sites [8], and the coordinating side-chains are conserved (Figure 2A). Based on these observations, the metals at these sites were assigned as calcium ions.

**Table 2:** Concentration (ppb) of metals in *S. pyogenes* Csn2 samples.

	Native Csn2	SeMet Csn2	Buffer
Ca	5.105×10^3^	8.579×10^3^	<0.3×10^3^
Mg	<5	<5	<5
Mn	11.9	8.0	10.3
Co	<5	<5	<5
Ni	20.71	5.63	<5

Although the metal analysis showed that significant amounts of calcium ion were present in the *S. pyogenes* Csn2 samples, it is still possible that the calcium ions were replaced with other metals during crystallization. To verify our metal assignment, we have replaced the calcium ions in the structure with other ions (sodium and potassium) or water molecules, re-refined the structures, and analyzed the coordination distances and B factors (Table 3). The resulting bond distances disfavor the inclusion of potassium ions in the sites because differences from the average distances observed in the Protein Data Bank were much larger than with other ions [9]. The refinement with potassium ions also resulted in unreasonably high B factors. In contrast, sodium ions and water molecules yielded lower B factors relative to their coordinating atoms, which could not be compromised by adjusting occupancies. Calcium ions resulted in B factors that were ∼11 Å^2^ larger than their coordinating oxygen atoms, which is not uncommon at this resolution [9]. Approximately 25% of the calcium ions found in protein crystal structures at a resolution of ∼2.2 Å in the Protein Data Bank display differences of greater than 10 Å^2^ between their B factors and the mean B factors of their coordinating atoms [9]. It was therefore considered acceptable to model calcium ions in these sites although we cannot completely exclude the possibility of heterogeneities such as lower occupancy of calcium ions and their partial replacement with sodium ions or water molecules.

**Table 3:** Comparison of *S. pyogenes* Csn2 structure with the metal identified as calcium, sodium, potassium or water.

Metal	Calcium			Sodium			Potassium			Water		
Monomer/Site	A/CA1	A/CA2	B/CA1	A/CA1	A/CA2	B/CA1	A/CA1	A/CA2	B/CA1	A/CA1	A/CA2	B/CA1
**R_cryst_/R_free_ (%)**	21.5/25.8			21.5/25.9			21.8/26.2			21.5/25.8		
**Metal B factor (Å^2^)**	63.24	62.20	59.03	44.32	42.27	39.59	101.62	71.56	79.21	26.86	27.88	24.05
**Average B factor of coordinating atoms (Å^2^)**	52.34	53.93	45.21	55.27	54.92	47.27	56.80	57.49	51.24	53.13	54.20	45.68
**Average distance of Metal-O(Protein) (Å)**	2.78 (2.43)	2.59 (2.43)	2.56 (2.43)	2.87 (2.50)	2.65 (2.50)	2.63 (2.50)	3.42 (2.74)	3.03 (2.74)	3.16 (2.74)	2.64	2.34	2.39
**Average distance of Metal-O(H_2_O) (Å)**	2.79 (2.49)	2.81 (2.49)	2.86 (2.49)	2.88 (2.58)	2.84 (2.58)	2.93 (2.58)	3.58 (2.72)	3.12 (2.72)	3.59 (2.72)	2.48	2.63	2.39

Values in parentheses are average distances observed in medium-resolution (2.0 to 2.5 Å) structures in the Protein Data Bank [9].

There are two different types of calcium-binding sites, CA1 and CA2, within or adjacent to the interface created by the two α-helical domains (Figure 3A). Six calcium ions (four in CA1 sites and two in CA2 sites) are present in the *S. pyogenes* Csn2 tetramer, compared with eight calcium ions per *E. faecalis* Csn2 tetramer [8]. Only three independent calcium ions were identified in the *S. pyogenes* Csn2 structure as the asymmetric unit has two Csn2 monomers.

**Figure 4:**
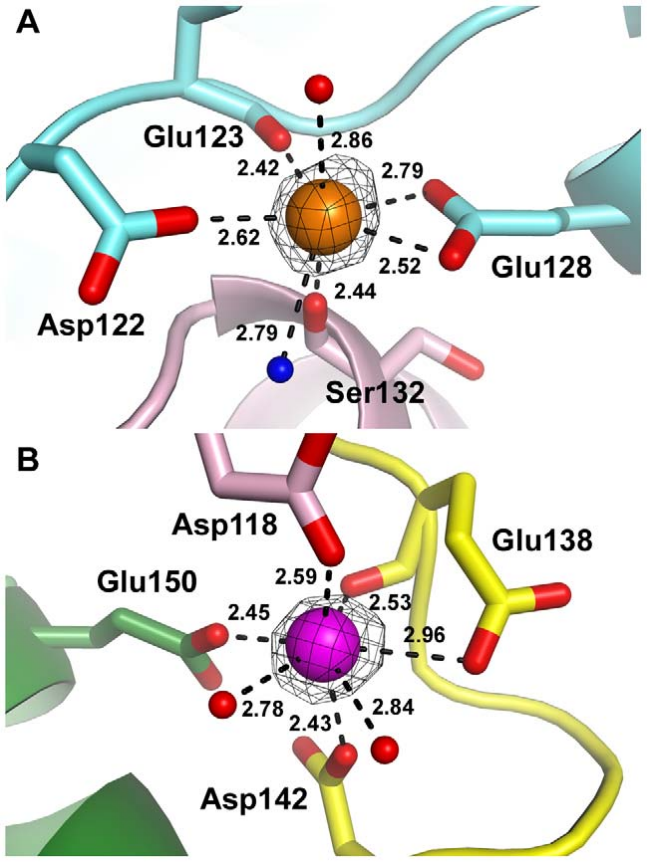
Calcium binding in *S. pyogenes* Csn2 structure. The CA1 (**A**) and CA2 (**B**) sites are colored as in Figure 2B, and coordinating oxygen atoms are shown in red. Among the two CA1 sites, the one adjacent to monomer B, which has lower B factors, is shown. The missing water molecule in the CA1 site, represented as a blue sphere, is modeled based on a comparison with the other CA1 site. Asp122 is shown in its calcium-binding conformation. The difference electron density map for the calcium ions was contoured at 10*σ*. The distances between the calcium ions and the coordinating atoms are also indicated.

The CA1 sites are located at the center of the interface formed by the α-helical domains of two interacting monomers. The calcium ions are coordinated by four amino acid residues (Asp122, Glu123, and Glu128 from one monomer and Ser132 from the other monomer) and one water molecule (Figure 4A). Asp122 residue was refined to have two different conformations, only one of which allows for calcium binding. The carboxylate group of Glu128 provides bidentate coordination to the calcium ion. Although each CA1 site has one coordinating water molecule, the placement of the water in the coordination geometry is different between the two binding sites. Considering the two independent CA1 sites together and the bidentate coordination by Glu128, it is likely that the CA1 sites employ incomplete pentagonal bipyramidal geometries in which one coordinating water molecule (an axial water in one CA1 site and an equatorial water in the other) was not modeled due to insufficient electron density. The multiple conformations of Asp122 and the missing water molecules suggest lower bound-calcium occupancy. However, the results of the metal analysis support full calcium ion occupancy in the CA1 sites of the selenomethionyl Csn2 protein used to determine the crystal structure (Table 2).

The only CA2 site in the asymmetric unit of the *S. pyogenes* Csn2 structure is located adjacent to one of the two hinge regions in monomer A. The calcium ion in the CA2 site is coordinated by three residues (Glu138, Asp142, and Glu150) from monomer A, Asp118 from monomer B of the symmetry-related dimer, and two water molecules (Figure 4B). Because both side-chain and main-chain oxygens of Glu138 participate in the calcium coordination at two equatorial positions, the CA2 site appears to have a distorted pentagonal bipyramidal geometry.

In the corresponding hinge region of monomer B in the asymmetric unit, we were unable to locate a calcium ion. In fact, the two monomers in the asymmetric unit have significantly different local structures in their respective hinge regions. In monomer B, Glu138 displays a completely different conformation compared to that of monomer A, and Asp142 was not modeled due to insufficient electron density. Furthermore, Asp118 in monomer A of the symmetry-related dimer exhibits a side-chain orientation different from that of the equivalent residue in monomer B, which participates in calcium coordination in the CA2 site. Based on these observations, we concluded that, indeed, no calcium ion was bound in the potential CA2 site adjacent to the hinge region of monomer B. This may indicate that the two types of calcium-binding sites, CA1 and CA2, have different affinities for calcium ions. The absence of calcium could also be a simple artifact caused by release of the calcium ion from the more exposed binding site during purification. It is important to note that the lack of a calcium ion is not a result of the selenomethionine (SeMet) labeling as our 2.9 Å native data also indicated the absence of calcium at this site.

In the study of *E. faecalis* Csn2, the authors found that its crystallization required the introduction of calcium ions, and that its behavior as a tetramer in size-exclusion chromatography columns was also dependent on the presence of calcium [8]. In contrast, we purified and crystallized *S. pyogenes* Csn2 without the addition of excess calcium ions. Despite this, the crystal structure of *S. pyogenes* Csn2 included bound calcium ions, suggesting their incorporation during protein expression. Surprisingly, the results of the analytical size-exclusion chromatography indicated that *S. pyogenes* Csn2 acted as a tetramer not only in the absence of added calcium but also in the presence of metal-chelating agents such as EDTA and EGTA (Figure 3B). This suggests that calcium binding may not be essential for the tetramerization of *S. pyogenes* Csn2. It is also possible that after the tetramerization is completed in the presence of calcium, the tetrameric structure remains stable even when EDTA or EGTA is added.

### Conformational changes in *S. pyogenes* Csn2

**Figure 5:**
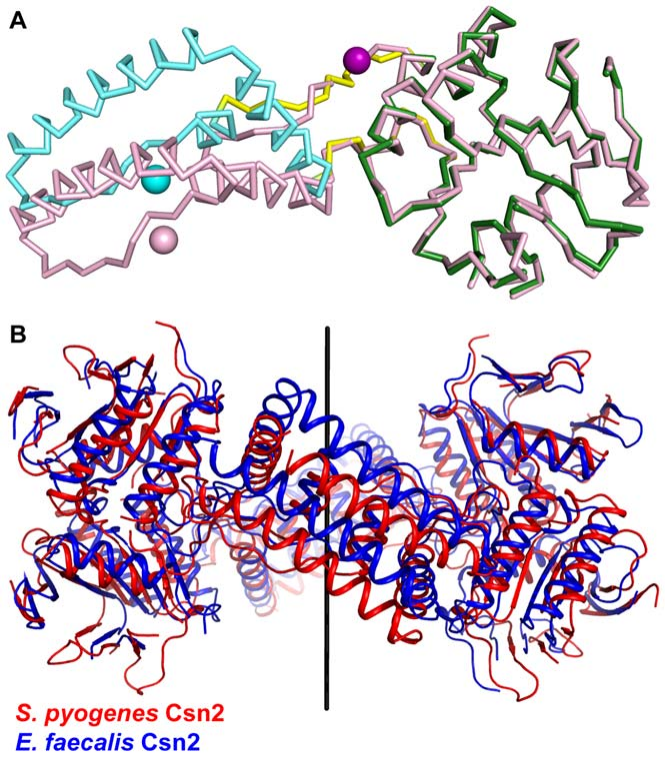
Conformational changes in *S. pyogenes* Csn2 structure. **A**: Structural alignment of *S. pyogenes* Csn2 monomers based on their α/β domains. Cα traces of the two monomers are colored as in Figure 2B except for the calcium ions in the CA1 sites of monomers A and B, which are shown in cyan and pink, respectively. **B**: Structural alignment of *S. pyogenes* and *E. faecalis* Csn2 tetramers based on their α/β domains. *S. pyogenes* and *E. faecalis* Csn2 tetramers are shown in red and blue, respectively. The two-fold symmetry axis is also indicated.

We structurally aligned the two *S. pyogenes* Csn2 monomers (monomer A and B), and noted a substantial deviation in the positioning of the two α-helical domains (Figure 5A). It appears that α2 can rotate, using its N-terminus as a fixed point. The distance and angle between the two α2 C-termini are approximately 13 Å and 27°, respectively. The two α3 helices and the following loops differ by up to 14 Å. The deviation between the two α4 helices decreases with increasing residue number, and the strain caused by the displacement of the α-helical domain is relieved by the flexible hinge region that connects α4 and α5.

The hinge region between α4 and α5 showed the most marked structural difference between the two Csn2 monomers. Comparing individual residues, both main-chain and side-chain conformations are completely different. This dissimilarity likely results from the difference in calcium binding. Residues in the hinge region of monomer A are structurally stabilized by the calcium ion bound in the nearby CA2 site, which is missing in monomer B. Such heterogeneity suggests that calcium binding in *S. pyogenes* Csn2 is important not only for its oligomerization, but also for its conformational diversity, enabling calcium-dependent conformational changes in the protein.

The presence of conformation-changing hinges within the Csn2 monomer was previously proposed in the study of *E. faecalis* Csn2, but its relationship to calcium binding was not considered [8]. Although calcium ions in the CA2 sites of *E. faecalis* Csn2 were also coordinated by residues in or adjacent to the hinge region [8], the lack of ‘calcium-deficient’ hinges in the structure made it difficult to recognize a connection between conformational change and calcium binding. In the *S. pyogenes* Csn2 structure, one of the two monomers in the asymmetric unit lacked a nearby CA2 site, which allowed us to detect the difference in the local structure of the hinge region, and the concomitant domain movement, upon comparison of the two monomers.

The conformational changes observed in *E. faecalis* Csn2 structure were subtle compared to those in *S. pyogenes* Csn2 structure. Although small structural variations were observed between the eight monomers in the asymmetric unit of *E. faecalis* Csn2 structure [8], their conformations were nearly identical to that of monomer A in *S. pyogenes* Csn2 structure. The root-mean-square-deviation (RMSD) values of the corresponding Cα atoms between *S. pyogenes* Csn2 monomer A and each of the eight *E. faecalis* Csn2 monomers range from 1.3 to 1.8 Å, whereas structural differences compared to *S. pyogenes* Csn2 monomer B are more substantial indicated by RMSD values ranging from 2.4 to 2.7 Å.

Despite the large conformational change between the two monomers, *S. pyogenes* Csn2 takes on a similar tetrameric ring shape to that of *E. faecalis* Csn2. Conservation of the positively charged inner surface of the ring in *S. pyogenes* Csn2 supports the previous proposition that Csn2 functions as a dsDNA-binding protein, accommodating its substrate through the center of the ring. The results of the electrophoretic mobility shift assay of *S. pyogenes* Csn2 also support its proposed dsDNA-binding activity (Figure 1). Although we cannot exclude the possibility of different binding modes, the results suggest that multiple *S. pyogenes* Csn2 tetramer rings can accommodate a single dsDNA molecule through their positively charged inner surfaces. This indicates a fast and continuous sliding motion of the Csn2 tetramers. It is not clear whether this multiple binding is physiologically relevant because the cellular concentration of Csn2 proteins may be significantly different and other Cas proteins may participate in the binding/sliding event.

Although the tetrameric ring shape is conserved between the *S. pyogenes* and *E. faecalis* Csn2 structures, notable conformational differences still exist between the two, presumably due to the distinctive conformation of *S. pyogenes* Csn2 monomer B (Figure 5B). We superimposed the *S. pyogenes* tetramer onto the *E. faecalis* Csn2 tetramer based on their α/β domains, and noted considerable structural deviation between their α-helical domains. Compared to *E. faecalis* Csn2, the structure of *S. pyogenes* Csn2 has translational displacement of the α-helical domain parts of the ring, while the rest of the ring is similar except for a slight twist. The translationally displaced part of the tetramer occurred nearly parallel to the two-fold symmetry axis going through the center of the ring. This type of movement is suggestive of accommodating a DNA double helix by sliding it through its inner opening.

It is not clear whether calcium dependence of the conformational change of Csn2 is physiologically relevant or not. It may simply have been a fortuitous revelation of the existence of different conformational states driven by crystallization. In fact, the residues that participate in the crystal packing interaction differ between the two monomers, although those that coordinate calcium ions are not directly involved (Table S1). Nevertheless, the crystal structure of *S. pyogenes* Csn2 revealed several interesting structural features of both the monomer and the tetramer. Further biochemical and biophysical analysis of *S. pyogenes* Csn2 will help clarify the role of conformational switching for its biological activity. In addition, elucidating its precise function, including its specific role in the CRISPR/Cas system, could lead to the development of novel antibiotics against the human pathogen, *S. pyogenes*. For example, inhibition of Csn2 function may reduce the pathogen's resistance to phage infection, and consequently, its viability [3].

## Materials and Methods

### Cloning, expression and purification

The *S. pyogenes csn2* gene was cloned into pHMGWA vector that contained a (His)_6_-maltose binding protein (MBP) tag and a tobacco etch virus (TEV) protease cleavage site [10]. This construct was used to transform *Escherichia coli* BL21 (DE3) cells. The transformed *E. coli* cells were cultured in LB medium at 37°C until the optical density at 600 nm reached 0.6. Then, protein expression was induced by the addition of 0.2 mM isopropyl-β-D-thiogalactopyranoside and incubation at 17°C for 18 hours. The cells were harvested by centrifugation and resuspended in lysis buffer (500 mM NaCl, 20% (w/v) glycerol, 5 mM β-mercaptoethanol (BME), 0.1% (v/v) Triton X-100, 10 mM imidazole, 0.25 mM phenylmethanesulfonyl fluoride, 20 mM sodium phosphate pH 7.4).

After cell lysis using a sonicator and centrifugation, the supernatant was loaded onto a 5 mL HisTrap HP column (GE Healthcare, USA) equilibrated with elution buffer (500 mM NaCl, 20% (w/v) glycerol, 5 mM BME, 20 mM imidazole, 20 mM sodium phosphate pH 7.4). After the column was washed with the elution buffer, the bound protein was eluted by a linear gradient of imidazole up to 500 mM, and dialyzed against TEV proteolysis buffer (500 mM NaCl, 20% (w/v) glycerol, 5 mM BME, 20 mM sodium phosphate pH 7.4). The N-terminal (His)_6_-MBP tag was cleaved by TEV protease and separated on another HisTrap HP column. The *S. pyogenes* Csn2 protein was further purified using a HiLoad 16/60 Superdex200 column (GE Healthcare, USA) equilibrated with size-exclusion chromatography buffer (500 mM KCl, 2 mM DTT, 5% (w/v) glycerol, 20 mM HEPES pH 7.5).

### Electrophoretic mobility shift assay

The binding of *S. pyogenes* Csn2 protein to dsDNA was tested by an electrophoretic mobility shift assay. DNA (150 ng) was incubated with *S. pyogenes* Csn2 protein in binding buffer (50 mM NaCl, 20 mM HEPES pH 7.5) at room temperature for 20 min. The molar ratio of DNA to Csn2 tetramer was 1∶0, 1∶1, or 1∶10. Reaction mixtures were separated in a 10% native Tris-glycine polyacrylamide gel, and analyzed after staining with ethidium bromide.

### Analytical size-exclusion chromatography

Analytical size-exclusion chromatography of *S. pyogenes* Csn2 was performed on a Superdex 200 10/300 GL column (GE Healthcare, USA). The column was equilibrated with buffer containing 200 mM NaCl, 2 mM DTT, and 10 mM Tris-HCl pH 8.0, and then 0.5 mL of 1.0 mg/mL *S. pyogenes* Csn2 was loaded onto the column at a flow rate of 0.4 mL/min. The experiments were repeated using buffer supplemented with 20 mM of CaCl_2_, EDTA, or EGTA.

### Structure determination

To determine a crystal structure of *S. pyogenes* Csn2, selenomethionyl protein was expressed in *E. coli* BL21 (DE3) cells grown in M9 medium supplemented with SeMet, as described previously [11]. The protein was purified as described above for native Csn2 protein. The selenomethionyl *S. pyogenes* Csn2 crystals were grown at 20°C by the hanging-drop method from 15 mg/mL protein solution in buffer (150 mM KCl, 4 mM DTT, 100 mM HEPES pH 7.0) mixed with an equal amount of reservoir solution (2.3 M sodium acetate pH 7.0). The crystals were cryoprotected in the reservoir solution supplemented with 20% (v/v) ethylene glycol, and flash-frozen in liquid nitrogen. Diffraction data for the selenomethionyl *S. pyogenes* Csn2 were collected at the AR-NW12A beamline of the Photon Factory at 100 K. The diffraction images were processed with HKL2000 [12]. Determination of selenium positions, density modification and initial model building were performed using SOLVE/RESOLVE [13], [14], [15]. The structure was completed using alternate cycles of manual fitting in COOT [16] and refinement in REFMAC5 [17] and PHENIX suite [18] with default geometry restraints. TLS refinement of four groups corresponding to individual domains in the asymmetric unit was also used. The stereochemical quality of the final model was assessed using MolProbity [19]. The atomic coordinates and structure factors were deposited in the Protein Data Bank [20] with the accession code 3TOC.

Native *S. pyogenes* Csn2 crystals were grown at 20°C by the hanging-drop method from 15 mg/mL protein solution in buffer (150 mM KCl, 4 mM DTT, 100 mM HEPES pH 7.0) mixed with an equal amount of reservoir solution (2.8 M sodium acetate pH 7.0). The crystals were cryoprotected in the reservoir solution supplemented with 20% (v/v) ethylene glycol, and flash-frozen in liquid nitrogen. Diffraction data for the native *S. pyogenes* Csn2 were collected at the beamline 6C of the Pohang Accelerator Laboratory at 100 K. The diffraction images were processed with iMOSFLM [21]. The selenomethionyl *S. pyogenes* Csn2 structure was used as a starting model for molecular replacement phasing in PHASER [22]. The structure was completed using alternate cycles of manual fitting in COOT [16] and refinement in REFMAC5 [17]. The stereochemical quality of the final model was assessed using MolProbity [19]. The atomic coordinates and structure factors were deposited in the Protein Data Bank [20] with the accession code 3V7F.

Sequence alignment was performed using ClustalW [23] and ESPRIPT [24]. For structural analysis and figure generation, the higher resolution selenomethionyl structure was used. Buried area calculation and molecular contact analysis were carried out using the CCP4i suite [25]. Hydrogen bonds were identified with PISA [26]. Figures were generated using PyMol (www.pymol.org).

### Metal analysis

The concentrations of five metals (Ca, Mg, Mn, Co, Ni) in *S. pyogenes* Csn2 samples were measured to reveal the identity of bound metals. The amount of calcium was determined using ICP-AES (Ultima 2C, Jobin Yvon, France), and concentrations of the remaining four metals were analyzed by ICP-MS (Elan 6100, Perkin Elmer, USA). Both selenomethionyl (3.3 mg/mL) and native (3.2 mg/mL) Csn2 proteins in buffer (150 mM KCl, 4 mM DTT, 100 mM HEPES pH 7.0) were analyzed with the buffer alone as a control.

## Supporting Information

Table S1 **Amino acid residues involved in crystal packing interactions.**
(PDF)
