# Supplementary material for: Crystal Structure of Streptococcus pyogenes Csn2 Reveals Calcium-Dependent Conformational Changes in Its Tertiary and Quaternary Structure
Source: PLoS One. 2012 Mar 30;7(3):e33401. doi: 10.1371/journal.pone.0033401 (PMC3316568; doi:10.1371/journal.pone.0033401)
Supplement: Table S1 — Amino acid residues involved in crystal packing interactions. (PDF) [file pone.0033401.s001.pdf]

Table S1. Amino acid residues involved in crystal packing interactions.[a]

Monomer A	Monomer B
Asn4	Arg15
Ser6	Phe47
Leu7	Lys50
Lys30	Mse51
Tyr34	Thr53
Glu43	Glu106
Lys45	Leu107
Phe47	Val109
Mse51	Phe110
Mse96	Glu111
Leu100	Glu146
	Glu183
	Tyr184
	Ser186
	Leu187
	Thr188
	Asn189

[a] Distance cut-off : 4.0 Å
